# Identifying ways of producing pigs more sustainably: tradeoffs and co-benefits in land and antimicrobial use

**DOI:** 10.1038/s41598-023-29480-5

**Published:** 2023-02-17

**Authors:** Harriet Bartlett, Andrew Balmford, James L. N. Wood, Mark A. Holmes

**Affiliations:** 1 Department of Zoology, University of Cambridge, Cambridge, CB2 3EJ UK; 2 Department of Veterinary Medicine, University of Cambridge, Cambridge, CB3 0ES UK

**Keywords:** Biodiversity, Antimicrobials

## Abstract

Pork accounts for the largest proportion of meat consumed globally and demand is growing rapidly. Two important externalities of pig farming are land use and antimicrobial resistance (AMR) driven by antimicrobial use (AMU). Land use and AMU are commonly perceived to be negatively related across different production systems, so those with smaller land footprints pose greater risk to human health. However, the relationship between land use and AMU has never been systematically evaluated. We addressed this by measuring both outcomes for 74 highly diverse pig production systems. We found weak evidence of an AMU/land use tradeoff. We also found several systems characterized by low externality costs in both domains. These potentially promising systems were spread across different label and husbandry types and indeed no type was a reliable indicator of low-cost systems in both externalities. Our findings highlight the importance of using empirical evidence in decision-making, rather than relying on assumptions.

## Introduction

Externalities are the positive or negative impacts of a system that not only affect those directly involved (e.g. the farmer). Livestock farming generates some striking externalities; whilst livestock production provides 30% of human dietary protein^1^, it occupies 75% of agricultural land^2^, emits 14–17% of anthropogenic greenhouse gas emissions^3,4^, and uses more antimicrobials than the whole of human medicine^5,6^. Demand for livestock products is high and rising^7^, especially for pork which has increased fourfold in the past 50 years^8^. How we meet this rising demand will be pivotal for health and the environment^9,10^. Livestock farming systems vary considerably in the scale of their externalities^11^, but our understanding of how multiple externalities co-vary across contrasting production systems is limited^12^. Research typically focuses on impacts in isolation, and the synergies or tradeoffs among them are extrapolated or assumed. To identify and promote the types of systems that best limit impacts or even carry co-benefits we need to explicitly consider multiple externalities^13^ and evaluate them across a wide range of alternative production systems.

Two important externality costs of livestock production are land use^14,15^ and antimicrobial resistance (AMR)^16^. Field studies of population densities of > 2500 individually-sampled species of vertebrates, plants and insects across five continents have consistently found that farming would have least impact on biodiversity if demand was met by coupling high-yield production systems with sparing remaining land for nature^17^. Whilst low-yielding systems may harbour more biodiversity on farm they, by definition need more land to produce a set amount of food, and the additional on-farm biodiversity in low-yield systems is not enough to counter the biodiversity loss from greater land use—farming land that could otherwise be spared for nature^17^. We therefore use land-use cost as a proxy for negative impact on biodiversity, with low land-use costs (high yields) representing lower impacts on biodiversity compared with a natural habitat baseline. There is also substantial evidence that land use co-varies with overall greenhouse gas emissions of contrasting production systems^12,18^, so systems with low land use also often have smaller greenhouse gas footprints. However, high-yield (i.e. low land-use) livestock farming raises important concerns. It is perceived to impose other negative costs including increased AMU which drives AMR^19–24^. Here we focus on AMU with primarily anti-bacterial properties as a proxy for risk of AMR^25–27^. AMU and hence AMR costs are often thought to be higher in “intensive” production systems^5,25,28,29^. Unhelpfully, “intensive” in this context is poorly defined. It is used to refer to varying dimensions of the farming system and is often used subjectively: for example, it is often defined by housing type or the level of inputs^30–32^. There is some agreement that agricultural intensification is linked to increased yields (production per unit area)^33^. It is therefore often stated that AMU and land-use costs trade off, so that interventions for mitigating AMU will probably exacerbate the damaging impacts of land use, and vice versa. However, data on yields (and hence land use) of livestock systems are patchy^12^, as is information on AMU^24,25,34–36^—and measures of both across a common set of production methods are very limited. In this study, we address these gaps by quantifying both externalities and how they co-vary for a broad range of pig systems in the UK which span the land-use costs^11^ and husbandry types of most commercial pig production systems across the world. Husbandry systems not included were those not permitted in the UK, such as those that use gestation crates. We aimed to characterise the relationship between these externalities. We also aimed to identify the types of systems that minimise externalities, grouping systems both by label type (taken here as membership of UK farm assurance schemes *Red Tractor, RSPCA-assured* and *Organic* and other labels free range and woodland; see “Methods”) and husbandry type for breeding systems (indoor, hybrid indoor-outdoor, and outdoor) and finishing systems (slatted, straw yard or outdoor).

We focus our analyses on pig systems as there is a pressing need to identify and promote systems that combine low externality costs. Demand for pork is increasingly rapidly^8^, pigs are the highest livestock users of antimicrobials globally^25^ and pork production uses ~ 120 million hectares of land including ~ 8.5% of global arable land^11^. To compare externality costs across alternative production systems, we estimated externalities as the sum of costs associated with the production of a unit of output^12^ (see “Methods”). We collected data from 74 UK breed-to-finish pig systems (which are responsible for 5–60% of the UK pig sector by label type; see “Methods”) and calculated their land-use and AMU costs. Land-use cost metrics are well established and reported in m^2^/kg deadweight (DW), including land used to rear animals and to produce feed. AMU cost metrics, however, are often reported using different, by necessity imperfect, proxies for AMR risk. Here we report data on three: mg/kg DW, mg/population correction unit (PCU), and defined daily doses (DDDvets). We reported these metrics as total use and for each of European Medicines Agency’s (EMA) four categories (A down to D) of importance for human and veterinary health^37^.

The aims of this study were to collect empirical data from real-world farming systems and use them to characterise the association between land-use and AMU costs. We also aimed to identify systems that carried low costs in both domains, if they existed.

## Results

All results are based on data collected from 74 commercial breed-to-finish systems with a range of label types. The label types can be approximately ordered by how demanding the required standards are, with higher categories exceeding the standards of lower categories. From lowest to highest the categories are: no certification or labelling ("none"), *Red Tractor* (including *Quality Meat Scotland; QMS*), *RSPCA assured,* free range, woodland and *Organic*. Systems can have multiple label types or none. If a system had multiple labels, we classified systems by the highest label type. Our sample consisted of: four systems with no label type, 31 *Red tractor*, 12 *RSPCA assured*, 18 free range, 3 woodland and 6 *Organic* systems. Of these 74 systems, 31 were indoor-bred, 2 were hybrid indoor/outdoor systems and 41 were outdoor-bred. 27 were slatted finished, 26 were outdoor-finished, 4 slatted and straw yard, 16 straw yards and 1 straw yard and outdoor. Some of the 74 systems were not independent of each other—they shared breeding and/or rearing farms. Where statistics are reported, this is for a subset of our farms with one randomly chosen from those that shared upstream farms (n = 43). Both externalities were not normally distributed (Shapiro–Wilk: land-use and antimicrobial-use costs both *p* > 0.01 and W = 0.70 and 0.68 respectively) so non-parametric tests were used.

### The land-use costs of contrasting systems

**Figure 1:**
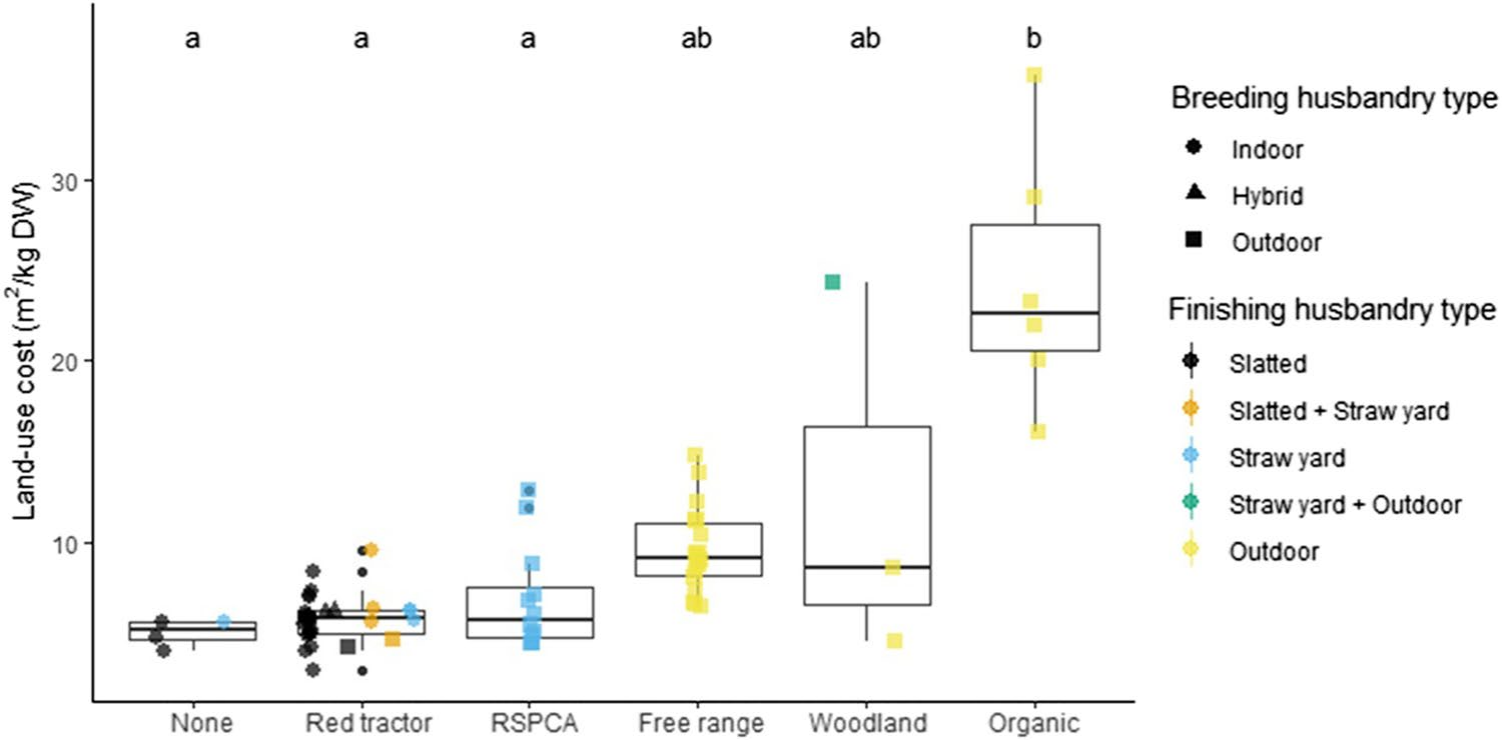
The land-use costs of breed-to-finish systems by label type, which includes land to rear animals and produce feed. The shapes and colours of scattered points show the husbandry type of breeding and finishing systems respectively. The letters above boxplots show the results from Dunn’s post-hoc tests, controlled for multiple comparisons using the Holm method, with different letters indicating significant differences between median values. The upper and lower hinges correspond to the first and third quartiles. Upper and lower whiskers extend to 1.5 times the interquartile range from upper and lower hinges respectively. The middle horizontal bar is the median. The smaller solid black dots refer to outliers which are any points that lie beyond the whiskers.

Land-use costs varied both within and between labelling categories and ranged from 3 to 36m^2^/kgDW and had significantly different medians (Kruskal–Wallis χ^2^ = 23.4, *p* < 0.01; Fig. 1). Post-hoc Dunn’s analyses revealed that *Organic* systems had significantly greater land-use costs than *RSPCA-assured, Red Tractor* and “none”. These land-use costs were estimated using economic allocation and FeedPrint^38^ country-specific yields, but system rankings were relatively insensitive to alternative allocation methods and sources of feed ingredient yields (see Supplementary Figure S1). There were also significant differences in land-use costs by both breeding and finishing husbandry types (Kruskal–Wallis χ^2^ = 12.1, df = 2, and χ^2^ = 21.4, df = 4 respectively, both *p* < 0.01), and post-hoc Dunn’s analyses found that outdoor-bred systems had significantly higher land-use costs than indoor-bred, and outdoor-finished than slatted and straw yard (see Supplementary Figure S2).

### The AMU costs of contrasting systems

**Figure 2:**
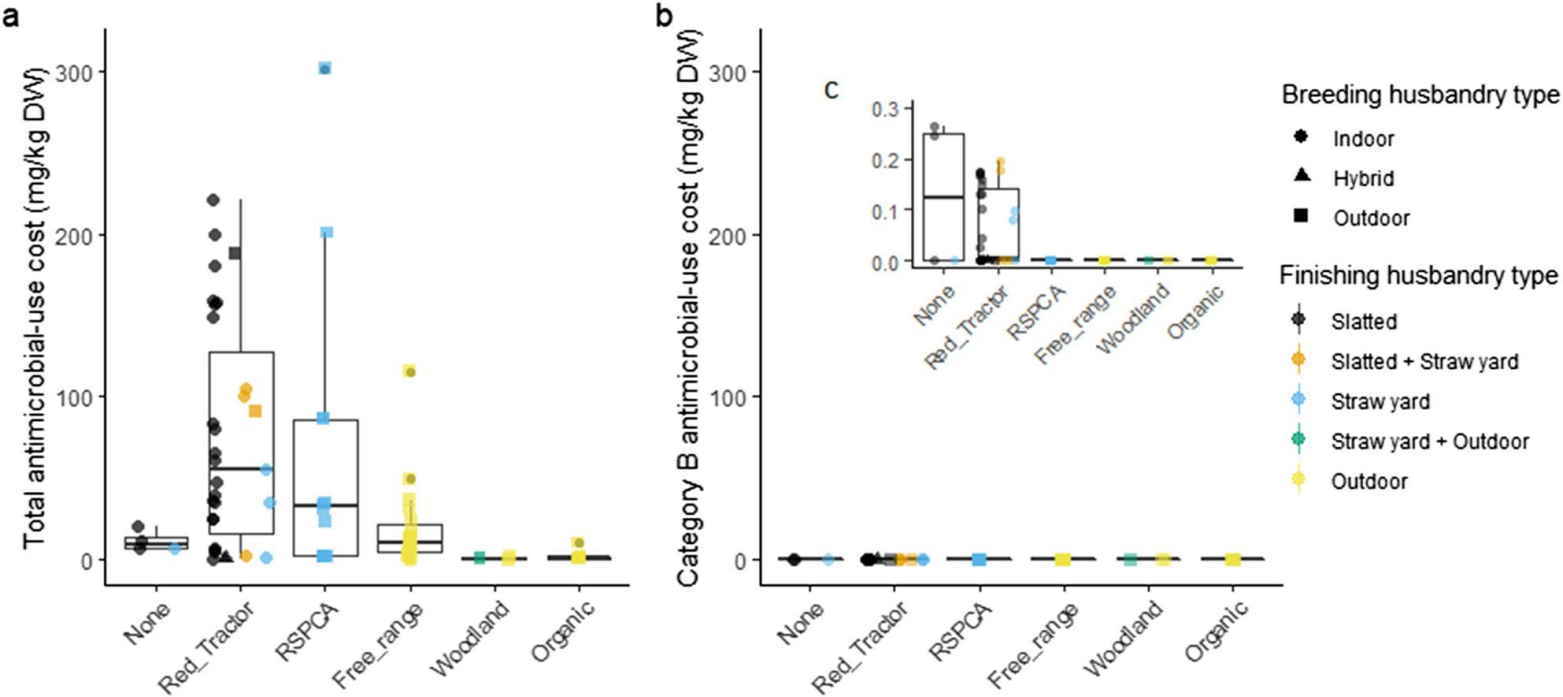
(**a**) total AMU costs and (**b**) category b AMU costs of breed-to-finish pig systems by label type and (**c**) an inset with adjusted y-axis scale. The shapes and colours of scattered points show the type of breeding and finishing subsystems respectively. The upper and lower hinges correspond to the first and third quartiles. Upper and lower whiskers extend to 1.5 times the interquartile range from upper and lower hinges respectively. The middle horizontal bar is the median. The smaller solid black dots refer to outliers which are any points that lie beyond the whiskers.

AMU costs ranged from 0 to 301 mg/kg DW (Fig. 2). Label types had significantly different median total AMU costs (Kruskal–Wallis χ^2^ = 11.68, df = 5, *p* = 0.04) but did not have significantly different Category B AMU costs (*p* = 0.11). Post-hoc Dunn’s analyses found no significant pairwise differences in total AMU costs. Note that we focus here on total and category B AMU in mg/kg DW as none of our studied farms used category A antimicrobials, and total, category C and category D use in mg/kgDW, mg/PCU and DDDvets were strongly intercorrelated (see Supplementary Table S1). There were significant differences in total and category B AMU costs by breeding husbandry type (Kruskal–Wallis χ^2^ = 9.5, χ^2^ = 10.9, *p* = 0.03 and *p* < 0.01 respectively, both df = 2) but not by finishing husbandry type. Post-hoc Dunn’s analyses found that indoor-bred systems had significantly higher category B AMU costs than outdoor-bred (see Supplementary Figures S3 and S4).

### AMU and land-use costs compared

**Figure 3:**
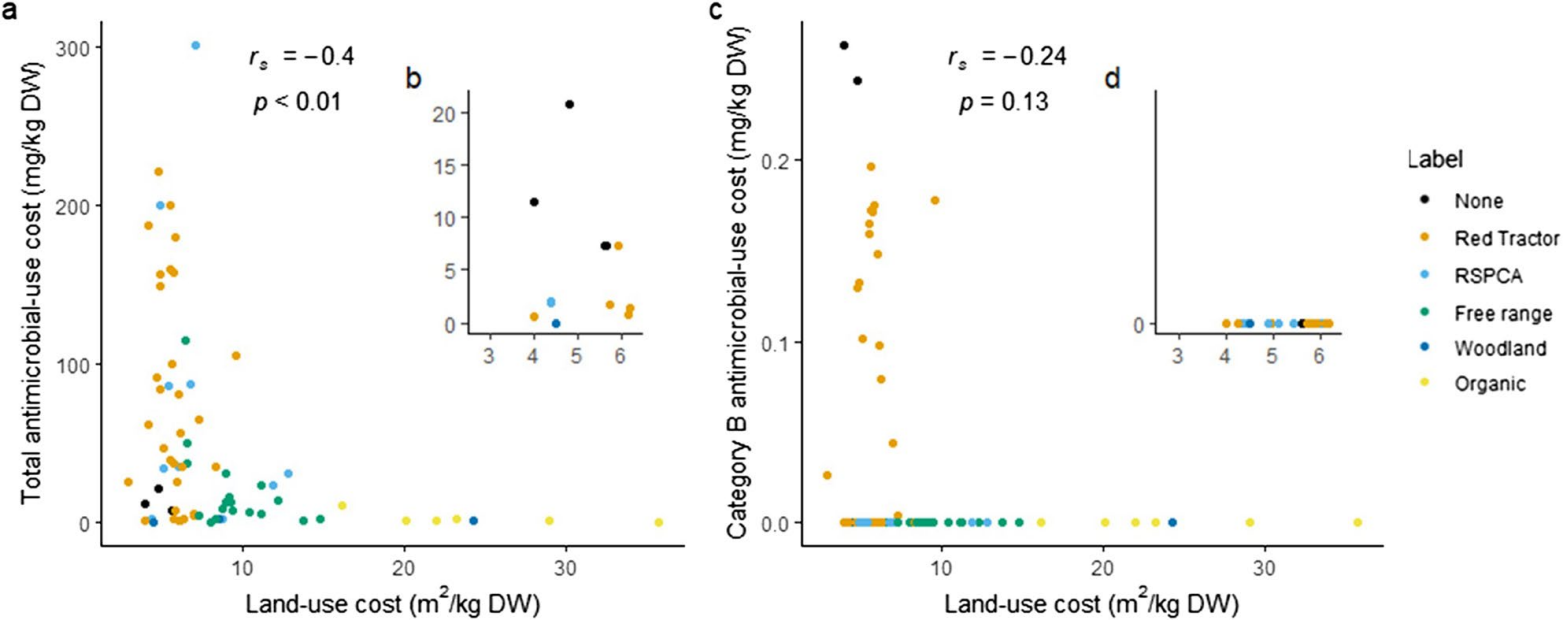
Land-use costs and (**a**) total AMU costs, (**c**) category B AMU costs and (**b**) and (**d**) insets showing systems in the top 50% for both costs. Colours indicate the different label types. r_s_ and *p* values are from Spearman rank correlations. Inset figures show systems scoring in the top 50% for both costs on each plot.

We did not find strong evidence of negative correlations between either total or category B AMU costs and land-use costs (Fig. 3). No label types performed poorly in both aspects (so there were no systems in the top right of the Fig. 3 plots), but many performed poorly in one. The best performing 50% of farms for both costs (insets, Fig. 3b and d and Supplementary Figure S5) still included systems from several label or husbandry types. The 11 breed-to-finish systems in the lowest 50% of breed-to-finish systems for land-use and both AMU costs (total and Category B) comprised eight *Red Tractor* systems (out of 61 in our sample), including three that were also *RSPCA assured* (out of 31), in addition to two (of four) “none” and one (of three) woodland systems, which was also free range (of 26); none of the six *Organic* systems fell in this best-performing set of farms. In our calculations, we excluded land under tree cover from our estimate of land-use cost assuming that woodland pig production maintains biodiversity at an equivalent level to natural habitat as there is some evidence that pigs can have a positive effect on biodiversity under these conditions^39,40^. The high performance of this woodland system is dependent on this assumption; when land under tree cover is not excluded this system is no longer in the lowest 50% of systems for land-use cost. When classified by husbandry type, five of these 11 most promising systems were indoor-bred (out of 32 studied), four were outdoor-bred (out of 42 studied), and two (of two studied) were hybrid indoor-outdoor bred. Of these same 11, but by finishing system husbandry type, five were finished with slatted floors, five in straw yards and one was outdoor finished (out of 30, 16 and 26 studied, respectively). No label type was reliably associated with high performance in both domains.

## Discussion

There is a widespread perception that “intensive” livestock production systems, which tend to be relatively higher-yielding (and so have lower land-use costs), have higher AMU costs^5,25,28,29,41,42^. Here we tested this perceived tradeoff by quantifying these externality costs for a broad range of contrasting pig systems. We have three key findings. Tradeoffs, often assumed to be inevitable, do not occur consistently and several pig production systems had relatively low land-use and AMU costs. Systems that combined low costs occurred in very different label and husbandry types, from those with no assurance certification or labelling with indoor breeding and slatted finishing, through to a fully outdoor woodland farm. Such systems provide substantial promise, as the low externality costs they achieved are clearly not unattainable. These are all commercial without exceptional advantages. Future work should explore how these best performing farms achieve such low externality costs. We also found no evidence that a single label type was consistently associated with low land-use and AMU costs so labelling does not provide reliable information for informed decisions about these externalities.

Our sample is not necessarily representative of the UK pig sector as study farms are likely to be affected by volunteer bias. Moreover, our AMU data carry uncertainty, our comparisons of different systems are limited by sample size, and the scope for drawing inferences for pig production elsewhere is limited because our farms are subject to UK-specific regulations. We attempted to minimise the effect of these factors in the following ways. Sample bias was minimised by recruiting farmer types that might otherwise be under-represented with the help of industry professionals, researchers, social media and internet searches. Accurate externality-cost data is challenging to obtain, particularly for AMU. The UK provides a useful study system as AMU data is validated and reporting compliance is high. Inaccuracy in our data is impossible to quantify but is most likely due to a lack of reporting of incomplete use of antimicrobials—for example where records show complete use of a unit of antimicrobial when in reality some was not used and was discarded because it passed its expiry date. This could have resulted in small overestimates in our AMU costs, but underestimates are much less likely. This allows us to be confident in our identification of low-cost systems. Although only UK farms were studied, we covered most pig husbandry types worldwide. This is supported by the fact that our range of land-use results spans beyond the world’s top and bottom 5% of pig producers^11^. Our findings may be limited by our sample size, for example our small samples of some farm and label types might have limited our ability to detect differences between them. However is by far the largest study of its kind comparing these externalities, with exceptionally high coverage of UK pig production (5–60% depending on label type; Table 1), and the only study to compare so many different label and husbandry types.

**Table 1:** Description of the 74 breed-to-finish pig systems studied. If systems met the requirements for multiple label types, they were included in the highest relevant type (see Supplementary Figure S7 for a Venn diagram illustrating the overlapping label types for our 74 studied systems). Any relevant standards or guidelines can be found in the citations in the first column and relevant extracts of the standards can be found in Supplementary Table S2. Note that free range and woodland do not have specific guidelines in the UK. The UK pigs by label type column shows the percentage of the total slaughtered fattening pigs in the UK in 2021^43^ with each label type^44^. These sum to more than 100% as farms can have multiple label types. The pigs in this study column shows the annual slaughtered fattening pigs from our 74 systems, summed by label type, and rounded to the nearest 1000, and our estimate of the % of all UK slaughtered pigs belonging to that label type which they represent. In total, our study covers 5% of UK slaughtered fattening pigs.

Label type	Breeding husbandry type	Finishing husbandry type	Number of breed-to-finish systems	UK pigs by label type	Number of pigs in this study (% of UK total pigs)
None^45^	Typically indoors. Farrowing crates are permitted	Typically indoors. Fully slatted floors are permitted	4	5%	38,000 (7%)
Red tractor^46^ including QMS^47^	Typically indoors. Farrowing crates are permitted	Typically indoors. Fully slatted floors are permitted	31	95%	479,000 (5%)
RSPCA assured^48^	Farrowing can be indoors, but sows must be allowed to turn around at all times	Pigs must have access to unperforated floors and sufficient bedding	12 (of which 10 are also *Red tractor*)	Unknown	222,000 (unknown)
Free range	Always outdoors		18 (of which 15 are also *Red tractor* and *RSPCA assured*)	2.5%	165,000 (60%)
Woodland	Pigs are kept at least with partial tree cover, but farms could also include some indoor housing		3 (of which 2 are also free range)	Unknown	13,000 (unknown)
Organic^49,50^	Always outdoors		6 (of which 5 are also *Red tractor, RSPCA assured* and all 6 are free range)	0.6%	31,000 (47%)

While the evidence generated in this study is relevant for two of the most critical issues we face, AMR and land use, there are other important externalities to consider. For example, carbon footprints are thought to correlate with land use, but trade off with animal welfare—but again, this assumption is largely untested. This study illustrates the importance of using empirical evidence rather than relying on anecdotally supported assumptions. Given the unexpected findings of this study, we believe that this warrants the systematic testing of other assumed relationships among externalities. Here we help address some important knowledge gaps for the pork sector but the same must be done on a much larger scale, spanning other externalities and sectors.

## Materials and methods

### Farms

Ethical approval was given by the Human Biology Research Ethics Committee (application number 2018.22) at the University of Cambridge prior to commencement and all methods were performed in accordance with relevant guidelines and regulations. Before participating in the study, all farmers gave informed consent. We contacted 150 UK pig producers, by phone or email, and 44 participated in the study. We obtained contact information for farmers from industry professionals, researchers, social media and internet searches. Our dataset included breed-to-finish systems belonging to the following label types: the assurance schemes *Red Tractor*, *RSPCA assured* and *Organic*, and the non-assurance labels free range and woodland, as well as those with no assurance or labelling (“none”). *Red tractor* standards build upon minimum UK standards. They allow fully indoor production, the use of farrowing crates and slatted floors. The *RSPCA assured* scheme is animal-welfare focused. It does not allow restrictive farrowing crates that do not allow the sow to turn around and all pigs must have enrichment. UK *Organic* standards require organically grown feed, permanent access to pasture and that phytotherapeutic and homoeopathic products, trace elements, vitamins and minerals are used in preference to antimicrobials when treating disease. Free range, whilst not a formal assurance, refers to fully outdoor systems, and woodland farms keep pigs with at least partial tree cover. See Supplementary Table S2 for the relevant regulations and standards. Our sample comprised 74 breed-to-finish systems which span both breeding and fattening stages. Breeding farms produce piglets that remain on the breeding farm until weaning. At weaning piglets move to the fattening stage, which can take the form of either two stages (rearing and finishing farms) or a single stage (a fattening farm). These stages can exist on one site or span several. Several participating producers had multiple farms, so our final dataset consisted of 74 data points, each of which represented a breed-to-finish system with a unique finishing or fattening farm, but some shared breeding and/or rearing farms (see Supplementary Figure S6 for a visualisation of this). Our data points are therefore not entirely independent of one another, which we address in our formal analyses (see “Statistical analysis” below).

Each system was visited between September 2018 and December 2020. We conducted questionnaire-based interviews (lasting ~ 30–90 min) with farm managers to collect data for estimating land-use and AMU costs. Where upstream farms didn’t exclusively supply a single finishing or fattening farm or a finishing or fattening farm was supplied by multiple upstream farms, externality costs were allocated proportionately. We did this by calculating externality cost per pig leaving a breeding and/or rearing farm and multiplying this by the number of animals entering a finishing/fattening farm. Where a farm had a variable number of animals, externality costs were calculated proportionally for a farm at steady state.

### Denominators

Externalities are reported per unit DW or PCU. This was calculated using data obtained through a questionnaire (see Supplementary Materials) on productivity, animal numbers and deadweight output (or liveweight and dressing percentages) and included DW from finishing pigs and sows slaughtered averaged over a year. For metrics using economic allocation, sow DW was equated to finishing pig DW using mean prices between September 2018 to December 2020 from the UK Agriculture and Horticulture Development Board and a large British pork processor. For example, if sow DW was worth 20% finishing pig DW, then 1 kg of sow DW was treated as 0.2 kg of finishing pig DW. For mass allocation and gross energy, kilograms of DW from sows and fattening pigs were treated equally—so 1 kg sow DW is equivalent to 1 kg finishing pig DW.

### Land-use cost

We estimated land-use cost as the total area of land required to produce a kg of pork, in m^2^/kg DW. It includes land used to rear the animals and produce their feed. The amount of land required to rear animals was obtained via the questionnaire, or if the farmer did not know this it was calculated from a map. It is hypothesised that pigs in woodland can have a positive impact on biodiversity^39,40^. There is limited evidence to support this, so we cautiously assume that woodland occupied by pigs has the same biodiversity as that not occupied by pigs, so any land under tree cover is excluded from our estimate of land-use cost for those systems.

The amount of land required to produce feed was calculated using information on the amount of feed used and the composition of each feed. For most feeds, exact formulations were obtained from the farm managers or manufacturers, but these cannot be shared due to Intellectual Property constraints. For the small portion of feeds that we could not obtain formulations for (1.5% by mass of the total annual feed used in our 74 study systems), the most similar feed formulation was used instead. Feed ingredient yields were obtained from FeedPrint^38^. Where possible, country of origin-specific yields were used. Where this information was not available because the country of origin was unknown, we assumed the country of origin was the same as the most similar feed with a known country of origin. If there was not yield data available for the country of origin, yields from the closest country (geographically and in production system practices) were used. Some feeds contained synthetic amino acids, and for some feeds their contribution was only reported as an aggregate amount and not broken down by individual amino acids. For these, each feed was matched in amino acid composition to the most similar feed, using amino acid compositions of feed ingredients from Feedtable (https://www.feedtables.com) and making up the deficit with synthetic amino acids. Where *Organic* yields were not available in FeedPrint, the percentage difference in *Organic* versus conventional yields was applied to FeedPrint’s conventional yields. We used Moakes and Lampkin^51^ for wheat, barley, oats and beans, Hossard et al.^52^ for maize, and De Ponti et al.^53^ for soya and peas. Where feeds were produced on land producing co-products, economic allocation was used to assign land-use cost of the focal feed. To test the sensitivity of our methodological choices, we also calculated land-use costs using mass and gross energy allocation and global mean crop yields for major feed crops: barley, maize, oats, peas, rapeseed, rye, soya, and wheat^54^ (see Supplementary Figure S1).

### AMU cost

AMU cost was calculated from medicines records for the most recent year of available data obtained via the questionnaire. There are several contrasting metrics used to quantify AMU cost, and there is no consensus on which should be used. The denominator used depends on the purpose of the reporting but often includes a measure of liveweight “at risk” at the time of treatment. Whilst this may be useful when considering clinical dosage, it is less useful for the comparison of externality costs where it is important to consider the farm’s ultimate unit of production^12^, for example a kilogram of DW. In this context, the numerator should reflect the risk of AMR from that AMU. The choice of numerator presents an inescapable challenge of how to account for the differences between antimicrobials which vary in dose weight and importance to human and veterinary medicine. For each of the studied farms we calculated three of the most common AMU cost metrics: mg/kg DW, mg/PCU and DDDvets/PCU, using EMA methodology^55^. We reported each of these metrics as the total use, and then separately for each of the EMA categories of importance to human and veterinary health^37^. From most to least important these are: Category A, “Avoid”, for antimicrobial classes not authorised in veterinary medicine, but authorised in human medicine in the EU; Category B, “Restrict”, for the highest priority critically-important antimicrobials including quinolones, 3rd and 4th generation cephalosporins and polymyxins; Category C, “Caution”, for those antimicrobials deemed important, but where their use in veterinary medicine is considered to present lower risks to human health compared with Category A and B; and Category D, “Prudence”, where the risk to public health associated with veterinary use is considered low but unnecessary use should still be avoided^37^. Supplementary Table S1 summarises the effects of metric choice on system rankings.

### Statistical analysis

To test whether label and husbandry types had significantly different externality costs we used Kruskal–Wallis tests with Dunn’s post-hoc analysis controlled for multiple comparisons using the Holm method (see Figs. 1 and 2 and Supplementary Figures S2, S3 and S4). To explore associations between land-use cost metrics (see Supplementary Figure S1) AMU cost metrics (see Supplementary Table S1) and externality costs (Fig. 3 and Supplementary Figure S5) we used Spearman rank correlations. These statistical approaches require data to be independent, which was not the case for some of the studied farms which shared breeding or rearing farms. There were insufficient data to remove the effects of shared farms statistically, so where statistics are reported this is for a subset of our data (n = 43), with one datapoint randomly selected from those that shared breeding or rearing farms (Supplementary Figure S6). Statistical analysis and data visualisation was carried out in RStudio 4.1.1 using the packages “stats”, “FSA”^56^, “ggpubr”^57^, “rcompanion”^58^, “ggthemes”^59^ “patchwork”^60^ and “ggplot2”^61^.

## Supplementary Information

Supplementary Information 1.

Supplementary Information 2.

## Data Availability

All data generated or analysed during this study are included in this published article and its supplementary information files.
