# Supplementary material for: Identifying ways of producing pigs more sustainably: tradeoffs and co-benefits in land and antimicrobial use
Source: Sci Rep. 2023 Feb 17;13:2840. doi: 10.1038/s41598-023-29480-5 (PMC9938186; doi:10.1038/s41598-023-29480-5)
Supplement: Supplementary file 2 — Supplementary Information 2. [file 41598_2023_29480_MOESM2_ESM.docx]

Supporting Information

Text: Questionnaire

Please provide the following information/data for the most recent year of available data prior to the farm visit.

1. Was there anything unusual about the most recent year of available data (e.g. an exceptional infectious disease outbreak)?*

2. Average number of Sows

3. Average number of Gilts/replacements

4. Average number of Boars

5. Average number of piglets born alive per sow per litter

6. Total number of litters per year per sow

7. Total number of piglets weaned

8. Source or breeding animals

9. Average age breeding animals sourced at

10. Please describe the pig flow in your system – numbers of pigs bought and sold with ages

11. Average finishing pig liveweight at slaughter

12. Average finishing pig deadweight at slaughter

13. Total number of pigs finished

14. Total number of sows sent to slaughter

15. Average weight of sows at slaughter

16. Area of land used to rear pigs

17. Membership of farm assurance schemes or other labels

18. Sow mortality over year (%)

19. Pre-weaned mortality over year (%)

20. Post-weaned mortality over year (%)

21. Please share details of all feeds including

a. Total tonnage used of each feed

b. Formulation of each feed

c. Where feed ingredients were grown/obtained from

22. Total weight of straw used

23. Please share formal records of your antimicrobial use (such as your electronic medicines book submissions) or give permission for us to obtain these from your veterinary surgeon.

Note that if this questionnaire was conducted on other farm types (e.g. breeding only), this questionnaire was adapted to only include relevant questions.

*Only one farm reported an exceptional event, and gave data excluding this time. This still meant that they provided a full year of data.


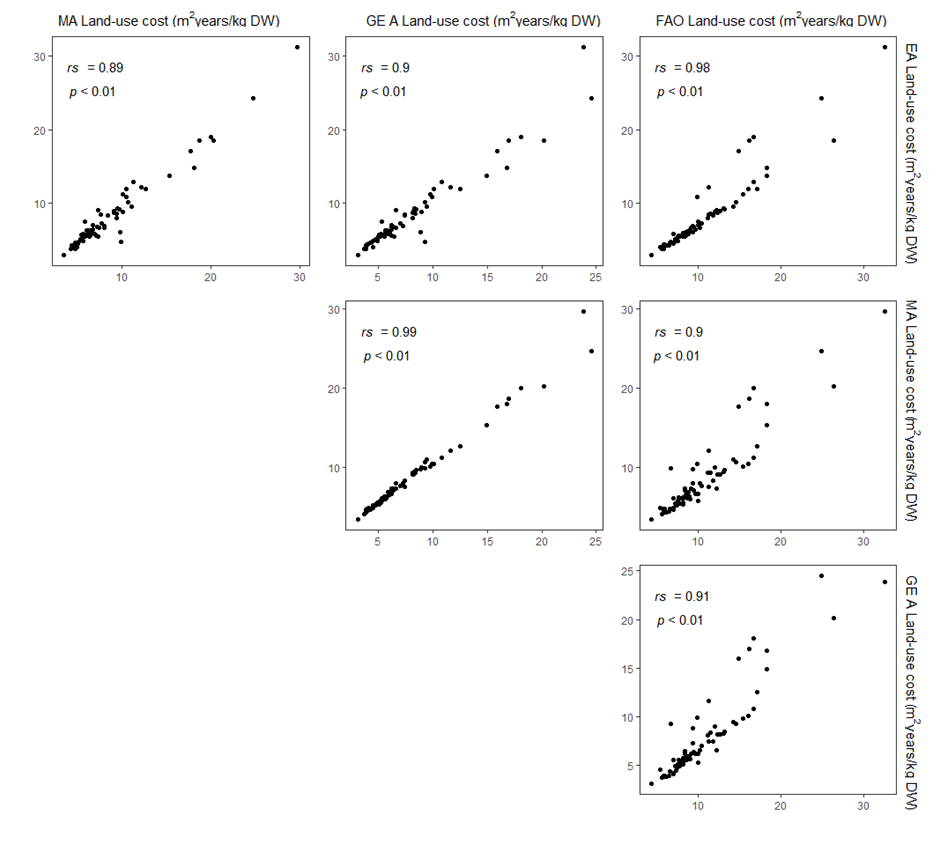

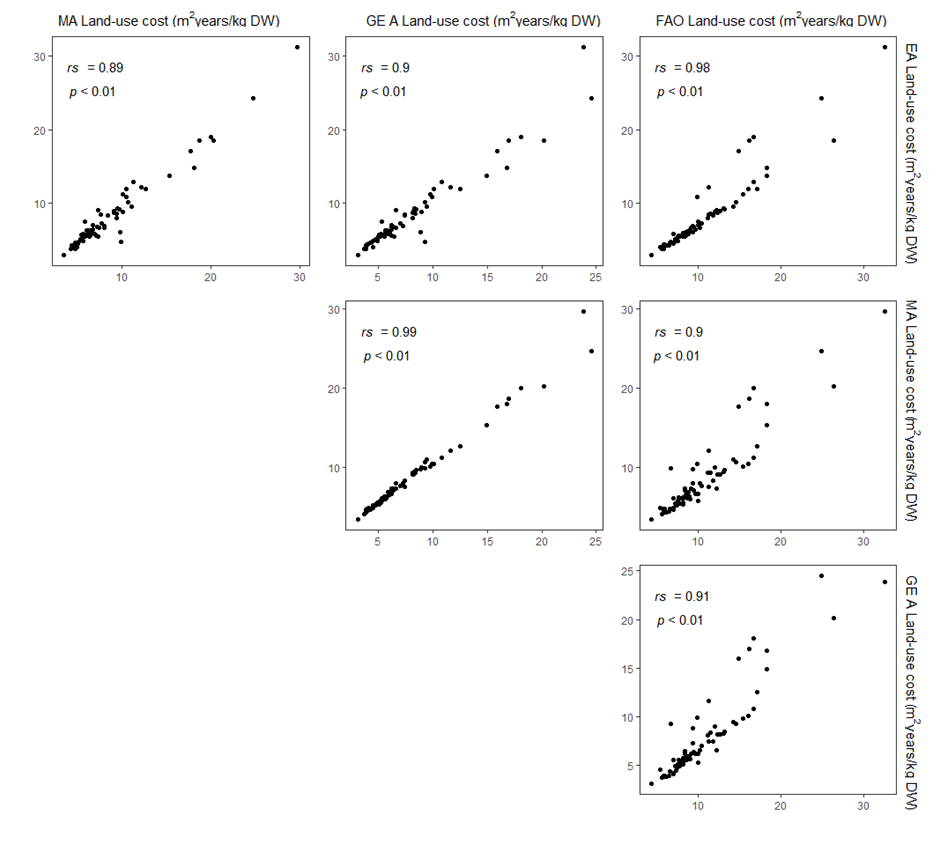

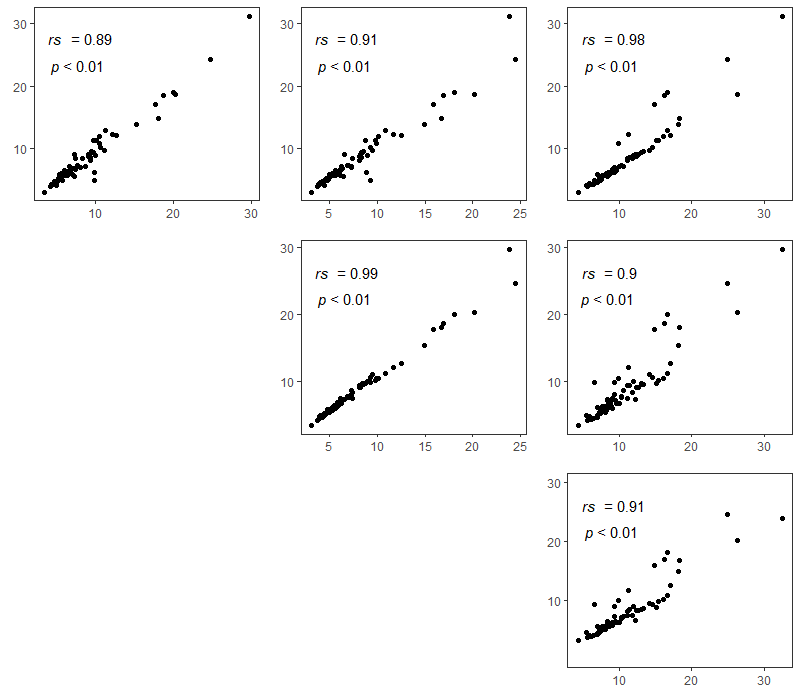


Fig. S1. Sensitivity analysis of land-use cost metrics. EA refers to economic allocation, MA is mass allocation, GE A is gross energy allocation, and FAO refers to a metric with economic allocation and global mean yields in 2020 from FAOSTAT for barley, maize, oats, peas, rapeseed, rye, soya, and wheat. rs and p values are from Spearman rank correlations.

Fig. S2. Land-use costs of breed-to-finish pig systems by a) breeding and b) finishing husbandry type. This includes land to rear animals and produce feed. The shapes and colours of scattered points show the husbandry type of breeding and finishing subsystems respectively. There were significant differences in land-use costs among husbandry types (Kruskal-Wallis: χ2= 11.1, df=2 and X2=23.3, df=4 for breeding and finishing systems respectively, both p<0.01). The letters above boxplots show the results from Dunn’s post-hoc tests, controlled for multiple comparisons, with different letters indicating significant differences between median values. Indoor-bred systems had lower land-use costs than outdoor bred (p<0.01), and outdoor-finished than straw yard (p=0.01) and slatted (p<0.01). The upper and lower hinges correspond to the first and third quartiles. Upper and lower whiskers extend to 1.5 times the interquartile range from upper and lower hinges respectively. The middle horizontal bar is the median. The smaller solid black dots refer to outliers which are any points that lie beyond the whiskers.


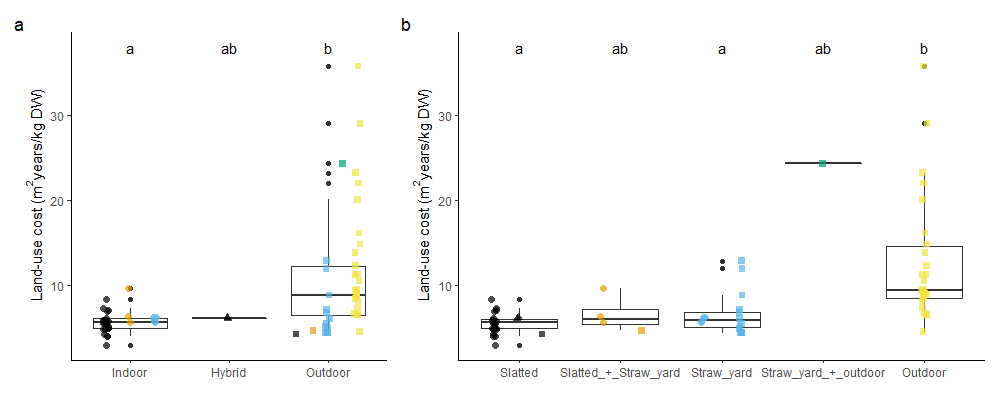

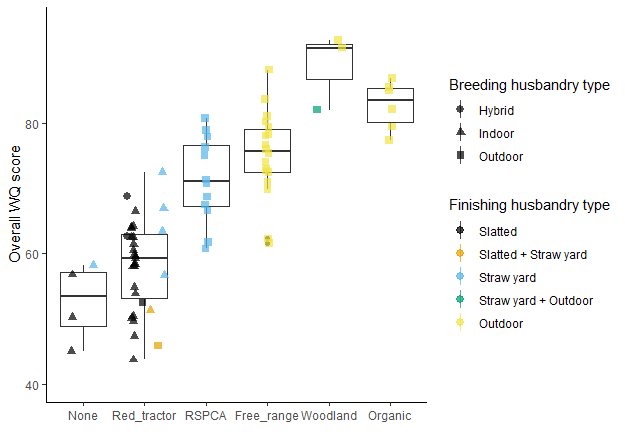

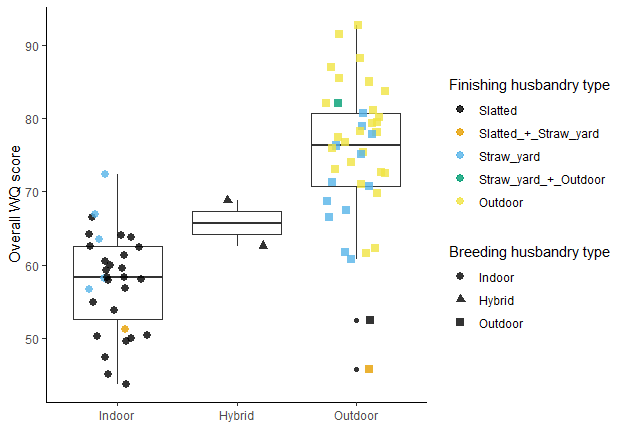

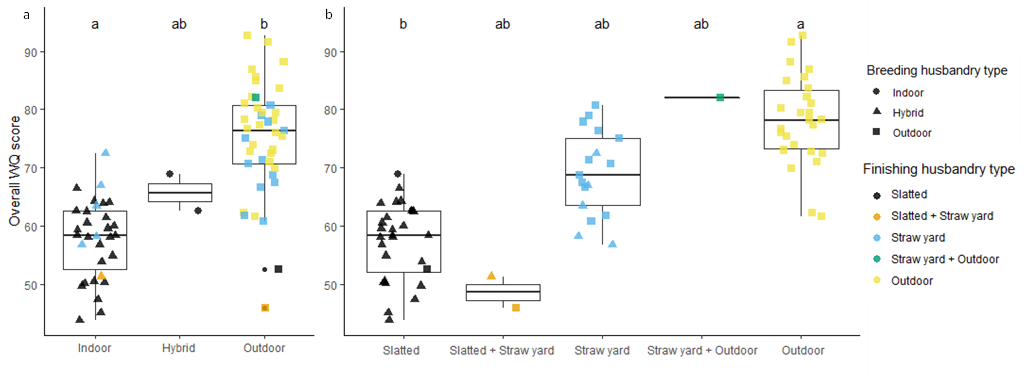


Fig. S3. Antimicrobial-use costs of breed-to-finish pig systems by a) breeding and b) finishing husbandry type. The shapes and colours of scattered points show the husbandry type of breeding and finishing subsystems respectively. There were significant differences between breeding husbandry types (χ2=7.31, df=2, p=0.03) but not finishing husbandry types (Kruskal-Wallis p>0.09). Post-hoc Dunn’s analyses did not find any pairs with significant differences. The upper and lower hinges correspond to the first and third quartiles. Upper and lower whiskers extend to 1.5 times the interquartile range from upper and lower hinges respectively. The middle horizontal bar is the median. **The smaller solid black dots refer to outliers which are any points that lie beyond the whiskers.**


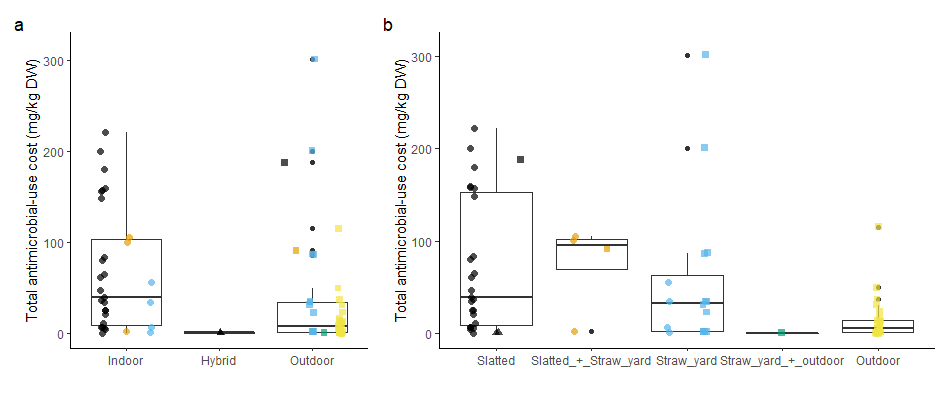

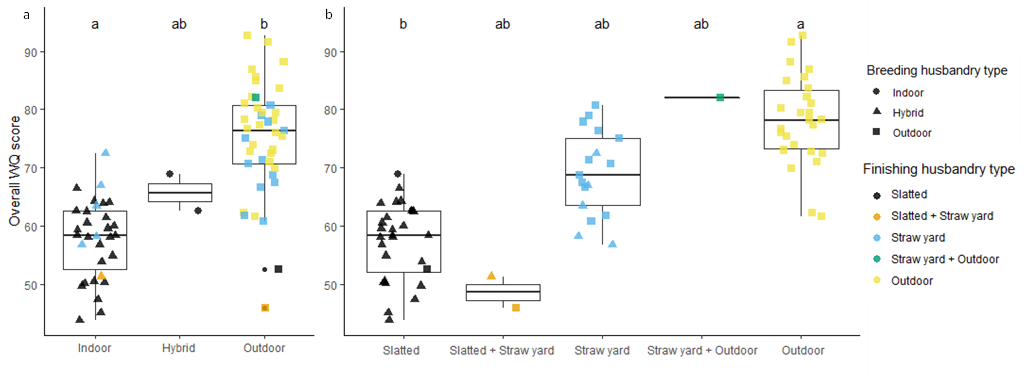

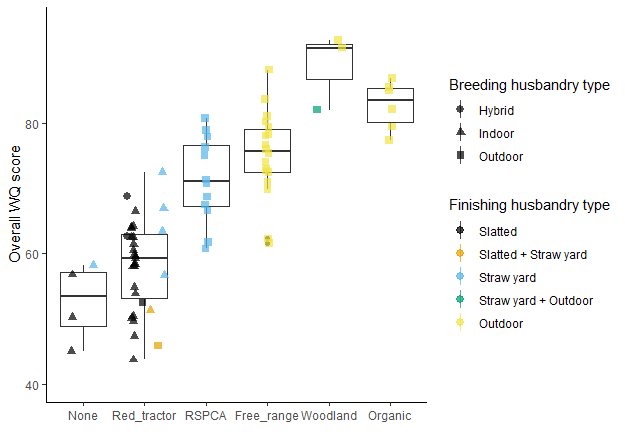

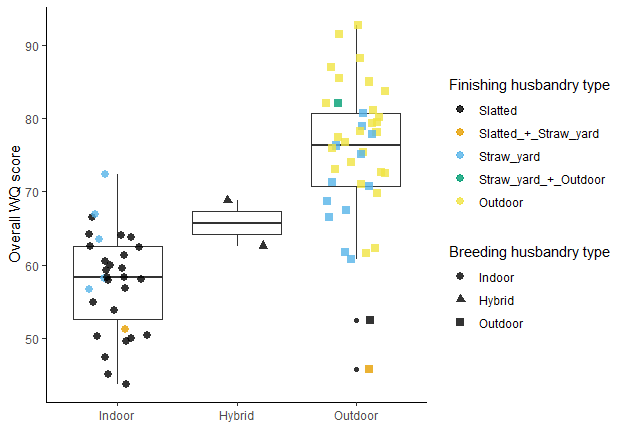


Fig. S4. Category B antimicrobial-use costs of breed-to-finish pig systems by a) breeding and b) finishing husbandry type. The shapes and colours of scattered points show the husbandry type of breeding and finishing subsystems respectively. There were significant differences between breeding husbandry types (χ2=10.9, df=2, p<0.01) but not finishing husbandry types (Kruskal-Wallis p>0.1). Post-hoc Dunn’s analyses found that Category B AMU cost was higher in indoor-bred systems compared with outdoor-bred systems (p>0.01). The letters above boxplots show the results from Dunn’s post-hoc tests, controlled for multiple comparisons, with different letters indicating significant differences between median values. The upper and lower hinges correspond to the first and third quartiles. Upper and lower whiskers extend to 1.5 times the interquartile range from upper and lower hinges respectively. The middle horizontal bar is the median. The smaller solid black dots refer to outliers which are any points that lie beyond the whiskers.


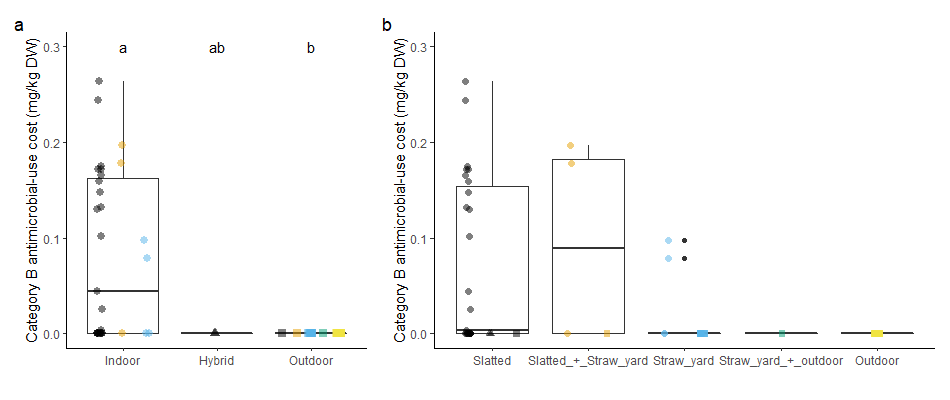

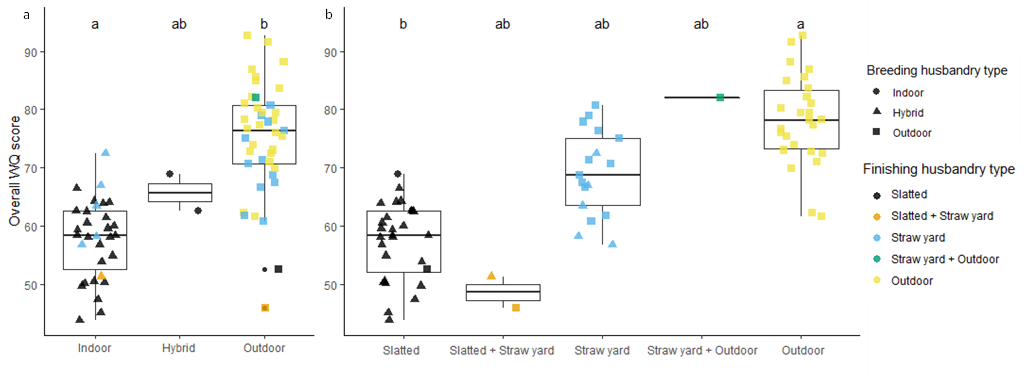

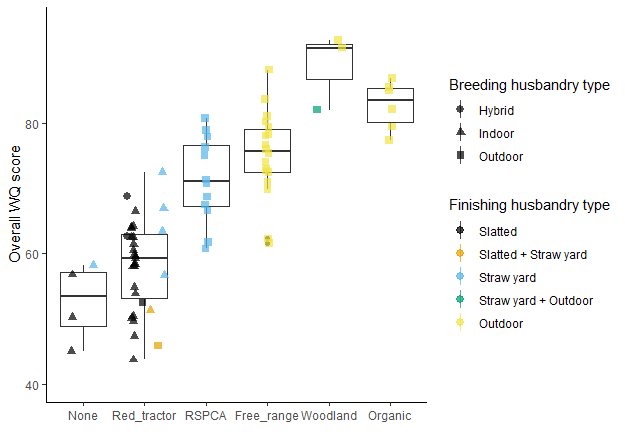

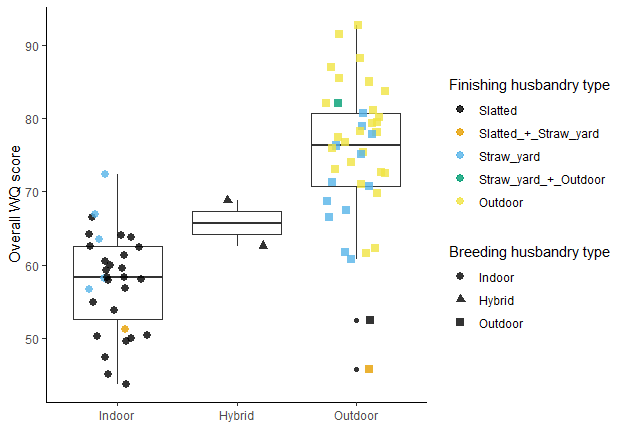


Fig. S5. Land-use costs and a) total antimicrobial-use costs and c) Category B antimicrobial-use costs of breed-to-finish systems. Colours indicate the different types of husbandry subsystems. rs and p values are from Spearman rank correlations. Inset figures b and d show systems scoring in the top 50% for both costs on each plot.


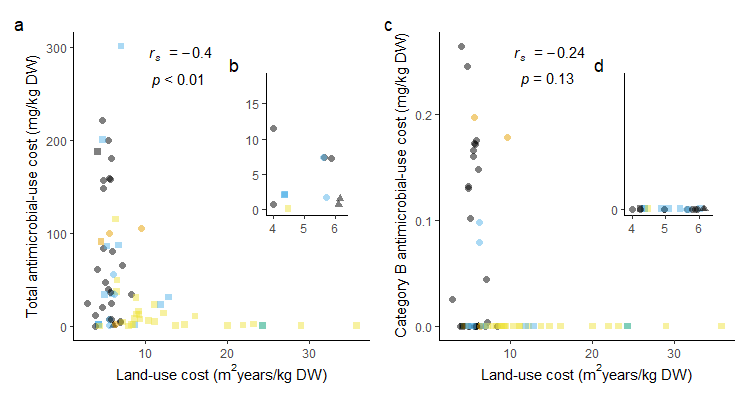

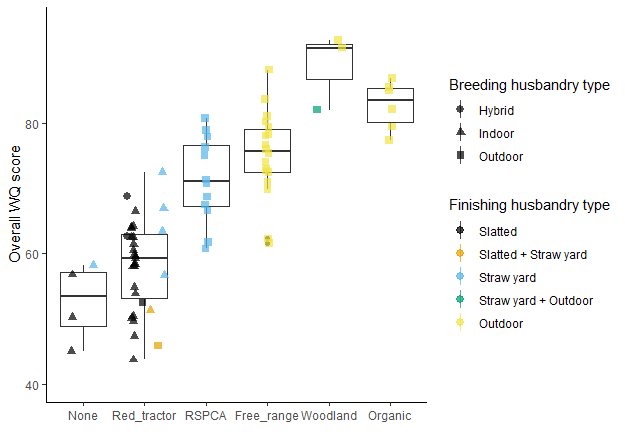

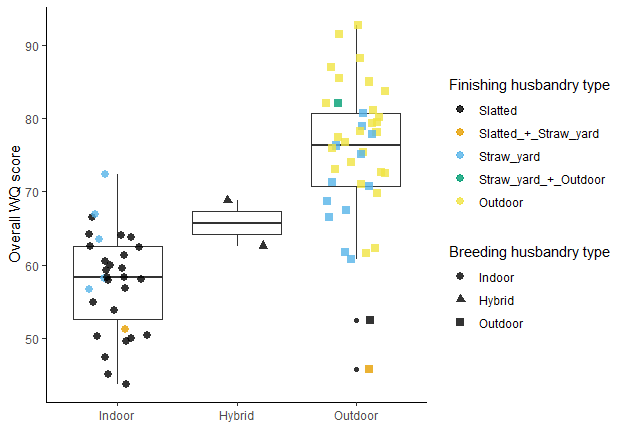


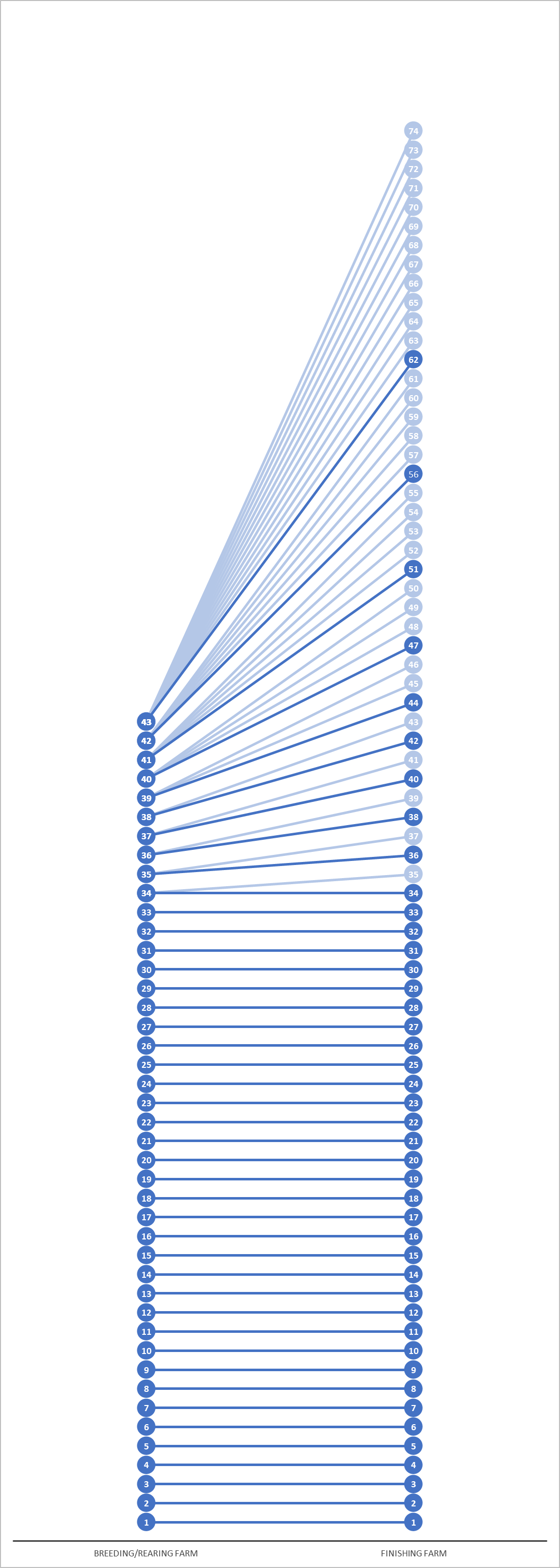


Fig. S6. Diagram of which finishing and fattening farms shared breeding or rearing herds. 33 breed-to-finish systems did not share breeding/rearing herds with another – shown by single horizontal lines from breeding/rearing farm to finishing farms 1-33. 41 breed-to-finish systems did share breeding and/or rearing herds – shown by the multiple lines from breeding/rearing farm on the left leading to the multiple finishing farms 34-74 on the right. In our statistical analysis, one finishing farm was randomly selected from those that shared breeding and/or rearing farms – as illustrated by the different shades of blue: farms included in statistical analyses are shown in dark blue and those excluded in pale blue. The numbers represent the number of breeding and/or rearing farms (in the left circles) and number of finishing farms (right circles) in this study. The lines between them show breeding farm supplied which finishing farm to illustrate how many of our sample shared breeding farms.


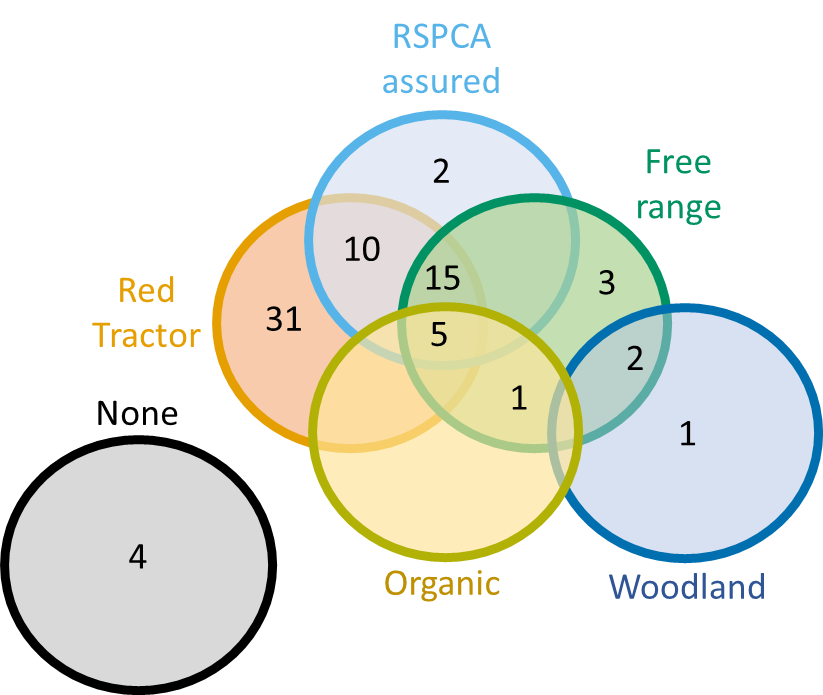


Fig. S7. Venn diagram to show the numbers of breed-to-finish systems in our study by label types. Those label types that overlap are layered according to their approximate degree of standards, with higher categories on top and lower categories underneath. From lowest to highest the categories are no certification or labelling ("None"), Red Tractor (including Quality Meat Scotland; QMS), RSPCA assured, free range, woodland and Organic. Note that one woodland farm has some indoor finishing, so is not included in free range. For our analysis we categorised systems by their most demanding label type. For example, of the 26 free range systems we profiled, 18 were assigned as free range, 2 as woodland and 6 as Organic.

Table S1. Spearman rank correlations between antimicrobial-use metrics in mg/kg DW, DDDvets and mg/PCU by EMA importance category (A down to D).

|  |  | mg/kgDW | | | DDDvets | | | | mg/PCU | | | |
| --- | --- | --- | --- | --- | --- | --- | --- | --- | --- | --- | --- | --- |
|  |  | C | D | Total | B | C | D | Total | B | C | D | Total |
| mg/kgDW | B | rs=0.44, p<0.01 | rs=0.40, p<0.01 | rs=0.39, p=0.01 | rs=1, p<0.01 | rs=0.35, p=0.02 | rs=0.36, p=0.02 | rs=0.37, p=0.02 | rs=1, p<0.01 | rs=0.44, p<0.01 | rs=0.40, p<0.01 | rs=0.40, p<0.01 |
|  | C |  | rs=0.72, p<0.01 | rs=0.81, p<0.01 | rs=0.43, p<0.01 | rs=0.96, p<0.01 | rs=0.73, p<0.01 | rs=0.82, p<0.01 | rs=0.44, p<0.01 | rs=1, p<0.01 | rs=0.72, p<0.01 | rs=0.90, p<0.01 |
|  | D |  |  | rs=0.98, p<0.01 | rs=0.40, p<0.01 | rs=0.73, p<0.01 | rs=0.99, p<0.01 | rs=0.96, p<0.01 | rs=0.40, p<0.01 | rs=0.74, p<0.01 | rs=1, p<0.01 | rs=0.97, p<0.01 |
|  | Total |  |  |  | rs=0.38, p<0.01 | rs=0.81, p<0.01 | rs=0.97, p<0.01 | rs=0.98, p<0.01 | rs=0.39, p=0.01 | rs=0.83, p<0.01 | rs=0.97, p<0.01 | rs=0.97, p<0.01 |
| DDDvets | B |  |  |  |  | rs=0.35, p=0.02 | rs=0.36, p=0.02 | rs=0.36, p=0.02 | rs=1, p<0.01 | rs=0.44, p<0.01 | rs=0.40, p<0.01 | rs=0.40, p<0.01 |
|  | C |  |  |  |  |  | rs=0.76, p<0.01 | rs=0.85, p<0.01 | rs=0.35, p=0.02 | rs=0.96, p<0.01 | rs=0.72, p<0.01 | rs=0.80, p<0.01 |
|  | D |  |  |  |  |  |  | rs=0.97, p<0.01 | rs=0.36, p=0.02 | rs=0.75, p<0.01 | rs=0.98, p<0.01 | rs=0.97, p<0.01 |
|  | Total |  |  |  |  |  |  |  | rs=0.37, p=0.02 | rs=0.83, p<0.01 | rs=0.96, p<0.01 | rs=0.98, p<0.01 |
| mg/PCU | B |  |  |  |  |  |  |  |  | rs=0.44, p<0.01 | rs=0.40, p<0.01 | rs=0.40, p<0.01 |
|  | C |  |  |  |  |  |  |  |  |  | rs=0.74, p<0.01 | rs=0.82, p<0.01 |
|  | D |  |  |  |  |  |  |  |  |  |  | rs=0.98, p<0.01 |

Table S2. Assurance scheme standards relevant to antimicrobial- and land-use costs. These are direct quotes copied from the standards or regulations.

| Labelling category | Standards relevant to antimicrobial use | Standards relevant to land use |
| --- | --- | --- |
| None ^37^ | You must record the:   - number of mortalities found on each inspection - date you treated any animals - name and address of the supplier where you bought any medicines you used in treatments - identity and quantity of medicines used - animal or group of animals you treated   You must keep these records for at least 3 years and make them available to any authorised person who asks for them, eg from Defra, the APHA or your local authority. | Pigs must be able to stand up, lie down and rest without difficulty and able to see other pigs at all times. They must also have enough space for them all to lie down comfortably at the same time.  For pregnant gilts and sows you need to:   - give them enough nesting material in the week before expected farrowing time, unless it’s not technically possible with the slurry system used and - keep a clear area behind the sow or gilt to ease natural or assisted farrowing.   You must feed sows and gilts using a system that makes sure that each individual can get enough food even when it has competitors for the food and give all dry pregnant sows and gilts enough bulky or high-fibre food, as well as high-energy food, to satisfy their hunger and need to chew.  You must make sure that all pigs, including sows and gilts, have enough manipulable material (such as straw, hay, wood, sawdust, mushroom compost, peat, or a mixture) for pigs to investigate.  For sows and gilts: The pen where the group is kept must have sides at least:   - 2.4m long - 5 or fewer pigs - 2.8m long - 6 or more pigs   You must offer a minimum unobstructed floor area. When working out the unobstructed area measurement you need to subtract any furniture that stops pigs from using the area above it.   \| **Pig** \| **5 of fewer animals (square metres)** \| **Standard (6 to 39 animals) minimum unobstructed floor area (square metres)** \| **40 or more animals (square metres)** \| \| --- \| --- \| --- \| --- \| \| Gilt after service \| 1.8 \| 1.64 \| 1.48 \| \| Sow when sows or gilts are kept in groups \| 2.48 \| 2.25 \| 2.03 \|   You must reserve an area of continuous solid floor:   - at least 0.95 square metres for each gilt - at least 1.3 square metres for each sow - up to 15% for drainage openings   This is a regular pattern that can include a fully slatted floor and must:   - cover no more than 15% of the area - be at least 0.95 square metres per gilt - be at least 1.3 square metres per sow   You can’t use close confinement stalls for breeding sows as they’ve been banned in the UK since 1 January 1999.  **Weaners and rearing pigs minimum unobstructed floor area**  You must provide weaner or rearing pigs reared in a group with a minimum unobstructed floor area.   \| **Average weight of pigs in the group** \| **Unobstructed floor area (square metres)** \| \| --- \| --- \| \| 10kg or less \| 0.15 \| \| 10-20kg \| 0.2 \| \| 20-30kg \| 0.3 \| \| 30-50kg \| 0.4 \| \| 50-85kg \| 0.55 \| \| 85-110kg \| 0.65 \| \| Over 110kg \| 1 \|   You must feed pigs at least once a day.  When pigs are housed in a group each pig must have access to feed at the same time if they:   - don’t have continuous access to feed - aren’t fed by an automatic feeding system that feeds the animals individually |
| Red tractor ^38^ (including QMS, Quality Meat Scotland ^39^) | Antibiotic or hormonal growth promoters are not used.  At least one person who is responsible for overseeing use of medicines on the unit has undertaken training and holds a certificate of competence/attendance from training undertaken since January 2018.  Training course covers at least:   - medicine types - antibiotic classes - medicine handling, administration and storage - recording requirements - avoiding residues - antimicrobial resistance - responsible use of antibiotics   Medicated feed is kept in separate clearly labelled bulk storage or bags.  Purchase records for all veterinary medicines must be kept  Records detail:   - identity of medicine - quantity - date of purchase - supplier name and address - batch number(s) - and assigned bottle number if using for administration records - expiry date(s)   Medicine purchase records are kept for 5 years.  Medicated Feeding stuffs Prescriptions (MFSPs) are kept for 5 years.  Records must be kept of all administered veterinary medicines (paper and/or electronic)  Records detail:   - identity of medicine - date of administration - quantity administered - length of withdrawal period for meat - identification of the animal or group of animals administered - batch number or assigned bottle number linked back to purchase records - number of animals treated - date treatment finished - date animal becomes fit for human consumption - name of person administering medicine - reason for treatment.   Medicine administration records are kept for 5 years.  Collated antibiotic data must be reviewed at least annually with the designated vet.  As part of the review the vet makes recommendations for alternative disease prevention and control measures to minimise prophylactic use of antibiotics, where possible.  Persistent high users of antibiotics must develop and implement an Antibiotic Reduction Plan, in conjunction with their designated vet.  Use of HP-CIA antibiotics (i.e. those belonging to Category B “Restrict”, as defined by the European Medicines Agency), must only be as a last resort, under veterinary direction.  The vet must at all times prescribe antibiotics in accordance with the Pig Veterinary Society (PVS) Prescribing Principles for Antimicrobials, which reflect RUMA guidelines.  The producer must take ultimate responsibility for all antibiotic use on the unit. | The unobstructed floor area available to each growing pig is at least:   \| **Average liveweight (kg)** \| **Minimum total floor area per pig (m^2^)** \| \| --- \| --- \| \| <10 \| 0.15 \| \| 10.1-20 \| 0.20 \| \| 20.1-30 \| 0.30 \| \| 30.1-50 \| 0.40 \| \| 50.1-85 \| 0.55 \| \| 85.1-110 \| 0.65 \| \| >110 \| 1.00 \|   Pigs have enough space to allow all the animals to lie down at the same time.  Pens used to house a group of sows/gilts have sides longer than 2.8m, except where there are 6 or fewer sows/gilts in which case the sides are no less than 2.4m in length.  The total unobstructed floor area available to each gilt after service and to each sow when gilts and/or sows are kept in groups is at least 1.64m^2^ and 2.25m^2^ respectively.  For gilts after service and pregnant sows, a part of the total unobstructed floor area required (at least 0.952m^2^ per gilt and at least 1.3m^2^ per sow) is of continuous solid floor of which a maximum of 15% is reserved for drainage openings.  Adult and working boars are provided with a minimum unobstructed floor area of 6m^2^ for their own use.  Where boar pens are used for natural service, a minimum of 10m^2^ floor area is provided and pen design allows adequate movement.  Where a farrowing crate is used the sow can stand up and lie down at full length, but excessive movement is prevented.  Nesting material is provided regardless of the slurry system.  The diet is adequate and suitable for the production status and body condition of the animal, especially pregnant animals which need a high fibre diet to satisfy their appetite.  For outdoor pigs: Stocking density for breeding animals must not exceed 30 adult pigs per hectare |
| RSPCA assured ^40^ | During the quarterly veterinary visit the following must be recorded:  a) the medicines permitted on a unit and their basic use  b) permitted in-feed medication  c) permitted in-water medication  d) permitted routine injectable or oral doser medication  e) permitted vaccines used, which animals are to be treated, dose rating, timing of vaccine and the frequency and reason for use  f) assessment of basic welfare  g) assessment and justification of any potentially injurious husbandry procedures  h) training courses attended or required  i) assessment of the data from the Collection and Communication of Inspection Results (CCIR) or equivalent.  It is recommended the electronic Medicine Book for Pigs is used to record antibiotic use. | Pigs must be fed a wholesome diet which:  a) is appropriate to their species  b) is fed to them in sufficient quantity to maintain them in good health  c) satisfies their nutritional needs  d) is accessible each day, except when required by the attending veterinary surgeon.  Foraging substrate must:  a) be provided to all pigs  b) be topped up regularly (at least every 3 days), or feed pellets must be broadcast over the entire pen area at intervals of no greater than one week.  The minimum space allowances for growing pigs are as follows:   \| **Live weight (kg)** \| **Bedded lying area (m^2^)** \| **Total area (m^2^)** \| \| --- \| --- \| --- \| \| 10 \| 0.10 \| 0.15 \| \| 20 \| 0.15 \| 0.225 \| \| 30 \| 0.20 \| 0.3 \| \| 40 \| 0.26 \| 0.4 \| \| 50 \| 0.31 \| 0.47 \| \| 60 \| 0.36 \| 0.55 \| \| 70 \| 0.41 \| 0.61 \| \| 80 \| 0.45 \| 0.675 \| \| 90 \| 0.475 \| 0.715 \| \| 100 \| 0.5 \| 0.75 \| \| 110 \| 0.53 \| 0.80 \|   Growing/finishing pigs in groups in straw yards must be provided with at least the following space allowances:   \| **Weight (kg)** \| **Total minimum space (m^2^) required if mucked out at least monthly.** \| **Total minimum space (m^2^) required if bedding topped up regularly but not mucked out.** \| \| --- \| --- \| --- \| \| Weaners to 35 \| 0.45 \| 0.5 \| \| 36-50 \| 1.17 \| 1.3 \| \| 51-75 \| 1.35 \| 1.5 \| \| 76-95 \| 1.5 \| 1.67 \| \| 96-110 \| 1.54 \| 1.72 \|   For dry sows:  a) sows must be given a minimum total floor space of 3.5m^2^/sow for mature adults, and 2.5m^2/^gilt for first and second parity animals  b) the lying area must be a minimum of 1.5m^2^/sow for mature adults, and 1m^2^/gilt for first and second parity animals.  Adult boar pens must:   - 1. be of such dimensions so as to enable animals to turn round easily and lie fully stretched   2. have a total minimum lying area of 7.5m^2^.   Service pens must be at least 10.5m^2^ in area, in order to be large enough to allow courtship and mating.  At least 2kg of straw must be provided per sow 48 hours prior to the expected farrowing date in order to satisfy nest building behaviour.  Farrowing accommodation must allow sows to turn around easily at all times, without any hindrance from fixtures and fittings present in the farrowing pen.  Pigs must, at all times, have access to effective environmental enrichment materials in sufficient quantities to allow and encourage proper expression of rooting, pawing and chewing behaviours.  For any system of outdoor production (including free-range):  a) the lying area (shelters) must be a minimum of:  (i) 1.5m2/sow for mature adults, and  (ii) 1m2/gilt for first and second parity animals  b) breeding animals must be stocked at no more than 30 adult animals per hectare (i.e. paddock space).  The minimum space allowances for growing pigs in outdoor systems are as follows:   \| **Live weight (kg)** \| **Bedded lying area (m^2^)** \| **Total area (m^2^)** \| \| --- \| --- \| --- \| \| 10 \| 0.10 \| 0.5 \| \| 20 \| 0.15 \| 0.5 \| \| 30 \| 0.20 \| 0.5 \| \| 35 \| 0.23 \| 0.5 \| \| 40 \| 0.26 \| 0.73 \| \| 50 \| 0.31 \| 1.3 \| \| 60 \| 0.36 \| 1.5 \| \| 70 \| 0.41 \| 1.5 \| \| 75 \| 0.43 \| 1.5 \| \| 80 \| 0.45 \| 1.67 \| \| 90 \| 0.475 \| 1.67 \| \| 95 \| 0.49 \| 1.67 \| \| 100 \| 0.50 \| 1.72 \| \| 110 \| 0.53 \| 1.72 \|   For producers wishing to label their product as ‘free-range’, growing pigs must be provided with:  a) a minimum total space (i.e. paddock space) of at least 12m2/pig where accommodation is moved to new paddocks after each batch.  b) a minimum total space (i.e. paddock space) of at least 40m2/pig where accommodation is not moved to new paddocks after each batch.  Sites must not be used for farrowing paddocks for more than 2 years.  Land used for farrowing paddocks must not be used again for farrowing sows for at least 6 months. |
| Free range | No formal guidelines | |
| Woodland | No formal guidelines | |
| Organic ^41,42^ – showing standards from the soil association | When treating you must use phytotherapeutic and homeopathic products and the trace elements, vitamins and minerals listed in standard 3.10.14 in preference to chemically-synthesised allopathic veterinary treatment or antibiotics, provided that their healing effect works for the animal species and the condition you are treating.  Where these treatments are inappropriate or will not be effective to avoid suffering or distress of the animal, chemically-synthesised allopathic veterinary medicinal products or antibiotics may be used under the responsibility of your vet.  You must not use chemically synthesised allopathic veterinary medicines or antibiotics for preventative treatment in the absence of illness or surgical intervention.  You must not use substances to promote growth or production of your animals (such as antibiotics, coccidiostatics and other artificial aids for growth promotion purposes).  You must not use critically important antibiotics except when no other treatment would be effective. Where these antibiotics have been used you must have veterinary justification for their use available at inspection in one or more of the following forms:   - post-mortem reports - results from sensitivity tests - vet site visit reports - veterinary instructions.   You must not use colistin. | You must make sure that by the time your livestock operation reaches organic status, any land on your unit used for their grazing or feed is either organic or in-conversion.  For Soil Association organic production you must allow your pigs permanent access to pasture or vegetated range, unless the circumstances listed in 3.6.1.1 prevent this.  To comply with the 170kg of nitrogen/ha/year limit you must not exceed the following stocking densities.   \| **Livestock type** \| **Category** \| **Maximum stocking rate per hectare** \| \| --- \| --- \| --- \| \| Pigs \| 7kg <13kg \| 170 \| \|  \| 13kg<31kg \| 40 \| \|  \| 31<66kg \| 22 \| \|  \| 66kg>intended for slaughter \| 16 \| \|  \| Breeding sow before first litter \| 15 \| \|  \| Sow with litter up to 7kg \| 9 \| \|  \| Breeding boar 66kg-150kg \| 14 \| \|  \| Breeding boar >150kg \| 10 \|   If you use field shelters and pig arcs, they must be covered, bedded and provide the following minimum lying area:   \| **Class of pig** \| **Minimum lying area – covered and bedded for outside shelters (m^2^/head)** \| \| --- \| --- \| \| Breeding pigs \|  \| \| Farrowing sows with piglets up to 28 days \| 4.0 \| \| Dry sows and boars \| 1.5 \| \| Fattening pigs \|  \| \| Up to 30kg (and over 40 days) \| 0.30 \| \| Up to 50kg \| 0.40 \| \| Up to 85kg \| 0.65 \| \| Up to 110kg \| 0.80 \|   The outdoor area for pigs must allow your pigs to dung and root. For the purposes of rooting different substrates can be used.  When housing your animals you must give them at least the following space:   \| **Class of animal** \| **Lying area or indoor area m^2^ per head** \| **Outdoor exercise area required m^2^ per head**  **Excluding pasture** \| **Total m^2^ per head** \| \| --- \| --- \| --- \| --- \| \| Farrowing sows with piglets up to 40 days \| 7.5 \| 2.5 \| 10 \| \| Piglets  Over 40 days and up to 30kg \| 0.6 \| 0.4 \| 1.0 \| \| Fattening pigs \|  \|  \|  \| \| Up to 50kg \| 0.8 \| 0.6 \| 1.4 \| \| Up to 85 kg \| 1.1 \| 0.8 \| 1.9 \| \| Up to 110kg \| 1.3 \| 1.0 \| 2.3 \| \| Breeding pigs \|  \|  \|  \| \| Sows \| 3.0 \| 1.9 \| 4.9 \| \| Boars \| 6  If pens are used for natural service: 10m^2^/boar \| 8.0 \| 14  If pens are used for natural service: 18m^2^/boar \|   You must not use farrowing crates.  You must feed your livestock organic feed that meets their nutritional needs at all stages of their development.  For your pigs and poultry, you must ensure that:  a) roughage, fresh or dried fodder or silage is added to their daily ration.  b) at least 20% of their total diet comes from your own holding. Where this is not possible, you may use feed produced in the same region in cooperation with other organic farms or feed business operators.   \| Pigs \|  \| \| --- \| --- \| \| Class  Sow + 6 piglets \| Average daily DMI (kg) \| \| 4.50 \| \| Plus each extra piglet \| 0.40 \| \| Gilts \| 2.60 \| \| Weaners at nine weeks \| 1.00 \| \| Weaners at 25 weeks \| 2.65 \|   If you cannot source 100% organic feeds that meet the nutritional needs of your animals, you may feed pigs and poultry up to 5% non-organic protein feed.  This percentage must be calculated on an annual dry matter basis. At your inspection you must have records to demonstrate that you are unable to source an appropriate 100% organic or in-conversion ration and that you have not fed more than 5% non-organic protein feed.  When you use commercial, compounded or blended feeds they must be licensed by an organic certification body and comply with organic standards. |
